# Sexually dimorphic gene expressions in eels: useful markers for early sex assessment in a conservation context

**DOI:** 10.1038/srep34041

**Published:** 2016-09-23

**Authors:** Benjamin Geffroy, Florian Guilbaud, Elsa Amilhat, Laurent Beaulaton, Matthias Vignon, Emmanuel Huchet, Jacques Rives, Julien Bobe, Alexis Fostier, Yann Guiguen, Agnès Bardonnet

**Affiliations:** 1 INRA, UR1037 LPGP, Fish Physiology and Genomics, Campus de Beaulieu, 35000 Rennes, France; 2 INRA, UMR 1224 Ecobiop, Aquapôle, Pôle Gest’Aqua, Quartier Ibarron, 64310, Saint Pée sur Nivelle, France; 3 UPPA, UMR 1224 Ecobiop, UFR des Sciences de la Côte Basque, allée du parc Montaury, 64600, Anglet, France; 4 UMR 5110 CNRS - UPVD (CEFREM), Université de Perpignan, Bâtiment R, 58 Avenue Paul Alduy, 66860 Perpignan Cedex, France; 5 Onema, pôle Gest’Aqua, 65 rue de Saint Brieuc, 35042 Rennes Cedex, France; 6 INRA, 1224 (U3E), Pôle Gest’Aqua, 65 rue de Saint Brieuc, 35042 Rennes Cedex, France

## Abstract

Environmental sex determination (ESD) has been detected in a range of vertebrate reptile and fish species. Eels are characterized by an ESD that occurs relatively late, since sex cannot be histologically determined before individuals reach 28 cm. Because several eel species are at risk of extinction, assessing sex at the earliest stage is a crucial management issue. Based on preliminary results of RNA sequencing, we targeted genes susceptible to be differentially expressed between ovaries and testis at different stages of development. Using qPCR, we detected testis-specific expressions of *dmrt1*, *amh*, *gsdf* and *pre*-*miR202* and ovary-specific expressions were obtained for *zar1*, *zp3* and *foxn5*. We showed that gene expressions in the gonad of intersexual eels were quite similar to those of males, supporting the idea that intersexual eels represent a transitional stage towards testicular differentiation. To assess whether these genes would be effective early molecular markers, we sampled juvenile eels in two locations with highly skewed sex ratios. The combined expression of six of these genes allowed the discrimination of groups according to their potential future sex and thus this appears to be a useful tool to estimate sex ratios of undifferentiated juvenile eels.

To date, environmental sex determination (ESD) has been detected in many vertebrates including turtles, sphenodons, crocodiles, lizards and fish species^1^. Protecting endangered species with ESD from decline should necessarily take into account this particularity that ultimately influences adult sex ratio^2^. For instance, it has recently been emphasized that turtle conservation projects might benefit from the knowledge acquired on ESD to efficiently manage populations (shading clutches was effective in reducing nest temperature, producing more males without compromising the fitness or hatching success)^3^. Early assessment of the sex of future spawners is of prime importance in order to consider the whole population dynamic^4^,^5^, build reliable stock-recruitment models^6^,^7^ and potentially help producing the rarer sex^3^. In fish, as in all vertebrates, gonadal development from an undifferentiated stage to a differentiated testis or ovary is triggered and regulated by a complex cascade of tightly regulated genes^8^. Some of these genes display early sex dimorphic expression before the first sign of histological or morphological gonadal sex differentiation. Even if the underlying mechanism of ESD has just begun to be understood in a model species such as zebrafish^9^, the research into early gene gonadal expression is an extremely promising area of research for understanding the basis of sex determination in vertebrates^10^.

Eel sex is highly influenced by the environment^11^, although it is not yet known to what extent genetic factors contribute to the final sexual phenotype. In recent years, most anguillid eels have seen their population decline worldwide^12^,^13^, triggering the general concern of different conservation entities^14^. The three temperate eel species from the northern hemisphere are already on the red list of the International Union for the Conservation of Nature classified as critically endangered (European eel, *Anguilla anguilla*) or endangered (American eel *Anguilla rostrata* and Japanese eel *Anguilla japonica*)^15^. The observed decline in eel species has been linked to direct and indirect anthropogenic causes such as habitat loss, overfishing, pollution, climate change and parasites^16^,^17^. However, apart from anthropogenic causes, the difficulty of preserving eel stocks relies on the many particularities that characterize their life history traits, especially panmixia and ESD. Eel species are deemed to have a complex life-cycle. All eels have a catadromous life-history, are long-lived semelparous and most likely panmictic^18^. Individuals from a same species reproduce once and in one place only, the Sargasso Sea (recent evidence has shown the route undertaken by American eels,^19^) for both the American and the European eels and near the Western Mariana Trench for the Japanese eel^20^. Once hatched, the larvae (leptocephali) drift or actively swim toward the continental shelf, where they metamorphose into the glass eel stage. Then, a portion of individuals spread into the estuary (elver stage) and progressively colonizes the whole watershed (at the yellow eel stage, fully pigmented). Finally, all individuals metamorphose (silver stage) and migrate back to their reproduction site.

A global conservation plan is difficult to set up since freshwater eels belonging to the Anguillidae family spread over a broad geographic range representing over 150 countries^15^.

Decisions regarding European eel population management have been taken at national and international levels since 2007 (EU regulation No. 1100/2007). The European Union has established goals for stock recovery and the eel has been classified in appendix II of CITES. This regulation required all member states to establish eel management plans, with the main objective of allowing at least 40% of the silver eel pristine biomass to migrate back to sea. This pristine biomass refers to the best estimate of escapement that would have existed if no anthropogenic influences had impacted the stock. Although measures have been readily taken after the EC decision, the biomass of silver eels that migrated back to sea in 2012 was only 6% of the “pristine state”^12^. The EU regulation also encourages restocking (which designates young eel transfer). The EU decreed that 60% of the yield of juvenile eels should be targeted for restocking. Based on this EU regulation, about 39 million glass eels and 15 million yellow eels were stocked in 2013^12^. However, there is not yet a clear understanding of what will be the long-term consequences of massive juvenile eel transplantation on future adult sex ratios, considering that sex determination is density-dependent. In eels, the sex ratio happens to be site-specific, with rivers geographically close producing a majority of males or a majority of females^21^. Usually, a greater proportion of males being observed in the downstream part of the watershed (high density), while females prefer to inhabit the rivers upstream^22^. In all eel species investigated to date, sex determination is influenced by the density of individuals and males predominate in crowded areas^11^,^14^,^23^. Hence, modifying eel density for conservation purposes might produce unanticipated effects linked to ESD.

The fishing of adult silver European eels during the time they join estuaries for their continental reproductive migration has been banned in most places. Therefore, the assessment of sex ratio now has to be done through electro-fishing. However, this cannot be achieved efficiently, because large individuals prefer deep habitat where they are difficult to sample. One solution would be to sample eels when their preferred habitat is still toward shallow geomorphological units i.e. before they reach 25 cm. It is worth noting that eels do not exhibit secondary sex characteristics. Hence, before male silvering and reproductive migration (which occurs earlier in the male life-cycle than in the female, and at a smaller size), sex can only be determined through gonad examination. The undifferentiated state of the gonad last for a relatively long time, and first signs of sex differentiation occur only when the animal reach a size of between 25 and 28 cm (depending on the sampling location^24^,^25^). From this point, the gonads either directly differentiate into ovaries or pass through an intersexual stage to later develop as testes^25^.

As previously explained, there is growing interest in developing tools that will allow the early sexing of eels, when they still have good catchability and are concentrated in the downstream part of the watershed. We aimed to tackle this difficult problem by investigating if early gonadal expression of some differentially expressed mRNA involved in sex differentiation would be a useful method to correctly assess sex ratio in young eel stages.

A recent review^26^ suggested a conserved role of gonadal gene expression among fish and mammals during vertebrate gonadal sex differentiation. In the present study, we first investigated the expression of 7 genes involved in sex differentiation and/or determination in European eels sampled at different stages of gonadal development having a differentiated gonad. Based on these results, and using 5 of these genes, we explored whether this molecular sexing strategy would allow us to sex small eels (<25 cm, likely to be histologically undifferentiated) sampled from both male and female sex-biased populations; we discuss our findings in an applied conservation context.

## Materials and Methods

### Ethical Note

All works herein comply with current French national laws on the handling of animals and were approved by local ethics committee “CE 73 COMITE OISEAUX POISSONS, INRA, France”, research protocol STP-E 0912 and E 1519.

Experiment 1: Assessing genes differentially expressed between males and females.

### Animals

Most of the experimental animals and methods used have been previously described^25^. Briefly, a subsample of 70 eels reared in experimental condition from the glass eel stage in INRA facilities (Aquapôle, St Pée sur Nivelle) and that reached the critical size of 29 cm (between 2 and 3 years) were first anaesthetized with benzocaine (0.3 ml.l^−1^ of water) and then the dose was increased up to 2 ml.l^−1^ of water, until the animal was killed. The left gonad of each individual was immediately frozen in liquid nitrogen and stored at −80 °C for later RNA extraction, while the right gonad was fixed in Bouin’s fluid for further histological analysis of the gonadal stages.

### Gonadal stages

Gonadal stages have been previously described^25^. Briefly, males (M), females (F) and intersexual (I, *i.e*. gonads with both oocytes and spermatocytes, named Syrski organ^27^) gonads were all divided into 2 groups: M1, M2, F1, F2, I1 and I2, where “1” represent earliest stages of the group and “2” a more advanced stage (See Table 1 for the number of fish in each group).

### Genes selected from the transcriptomic analysis

**Figure 1:**
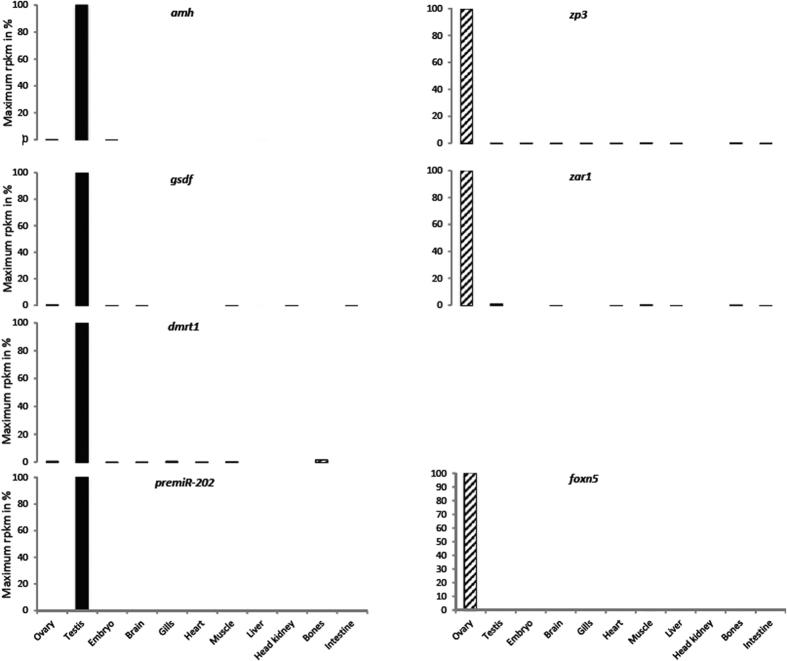
Gonad-specific expression of the 7 selected genes (*dmrt1*, *amh*, *gsdf* and *pre-miR202*, *zar1*, *zp3* and *foxn5*) detected through the RNA-seq analysis by the Phylofish program. Expression levels are expressed in reads per kilobase per million mapped reads (RPKM).

Seven genes were selected based on a multi-tissue RNA-seq analysis originating from the PhyloFish project^28^ (http://phylofish.sigenae.org/index.html) that aimed at characterizing the transcriptome of 11 different tissues/organs in a wide variety of teleostean and holostean species, including Europen eel (NCBI BioProject PRJNA256923). To generate the PhyloFish database, sequencing libraries were constructed from ovary and testis of one female (36 cm and 75.0 g) and one male (31 cm and 59.7 g), sampled at INRA aquaculture facilities in June 2011. Sequencing and *de novo* assembly were performed as previously described^28^. A total of 60,263 contigs were generated after *de novo* assembly. RNA-seq raw sequence data from the Hiseq2000 sequencer were deposited into NCBI SRA under accessions SRP045099. The Digital Differential Display (DDD) tool available in the PhyloFish browser was used to identify the most differentially expressed genes between ovary and testis. For further analysis, *dmrt1*, *amh*, *gsdf*, *pre*-*miR202*, *zar1*, *zp3* and *foxn5*, were chosen and primers designed using Primer3^29^ (see Table 2 for primer sequences and complete names of genes). Most of the genes selected were based on their RNA-Seq expression profiles (Fig. 1) and are well described in different animal taxa for being involved at different steps of gonadal differentiation and/or gonadal development.

For instance, zygote arrest 1 (*zar1*) and zona pellucida glycoprotein 3 (*zp3*) are only detected in growing oocytes of different fish species^30^,^31^, and thus their expression is specific of females even at early oogenesis stages. Concerning male-predominant genes, the DM (DNA-binding domain) related transcription factor 1 (*dmrt1*) is probably one of the most studied genes involved in testicular differentiation since, *dmy* is a duplicated paralog of *dmrt1* in the medaka, *Oryzias latipes*, and was shown to be the master sex-determining gene of that species^32^. In addition, this gene was shown to be involved in testicular differentiation in many different fish species, with a male-specific expression long before histological observation of male specific characteristics (*e.g*.^33^). The Anti-Müllerian hormone (amh), also known as Müllerian inhibiting substance (MIS), is known to play a key function in the control of germ cell proliferation and/or differentiation in future male gonads of different fish species^34^,^35^, including the Japanese eel (*Anguilla japonica*)^36^. The gonadal soma derived factor (*gsdf*) is predominantly detected in Sertoli cells surrounding spermatogonia in most fish investigated to date^37^,^38^ such as rainbow trout, *Oncorhynchus mykiss*, medaka (genetic sex determination) and zebrafish *Danio rerio* (polygenic sex determination^9^) and it acts to stimulate spermatogonia proliferation. The *foxn5* gene encodes a protein involved in different embryonic developmental processes such as early cleavage and gastrula stages^39^. Recently, a high expression level of *foxn5* was detected in ovaries of medaka when compared to testis^40^, suggesting that this gene is an essential transcript needed for oocytes or subsequent egg and embryo development. In human, fish and chicken, *mir-202* (or its homologues miR-202-5p and miR-202-3p) is highly differentially expressed between testis and ovaries^41^,^42^,^43^. These authors showed that the male-biased expression was noticeable at the onset of testicular differentiation^41^, which was also confirmed in mice, with high *mir-202* expression in spermatogonia, spermatocytes and spermatids^44^.

### RNA extraction and reverse transcription

Gonads were crushed with a disperser (Ultra-Turrax, IKA). Total RNA was extracted using Tri Reagent (Molecular Research Centre, Cincinnati, OH) according to the manufacturer’s instructions. An aliquot of each tube was diluted in diethylpyrocarbonate-treated water to reach the value of 180 ± 17 ng.μl^−1^ RNA. Then, 1.8 μg of RNA was reverse transcribed using 200 U Moloney murine Leukemia virus (MMLV) reverse transcriptase (Promega) and 2 μg random hexamers (Promega) in a master mix buffer and supplemented with 25 U of RNase inhibitor (RNasin; Promega) in a final volume of 25 μl. Reverse transcription products were then diluted 1:50 for quantitative real-time PCR (qPCR).

### Quantitative real-time PCR

RNA abundance analysis of genes encoding *dmrt1*, *amh*, *gsdf*, *pre-miR202*, *zar1*, *zp3* and *foxn5* was carried out using qPCR. Sixty-seven samples of cDNA were analysed using the Fast SYBR Green Master Mix (Applied Biosystem). Each well contained 4 μl of cDNA, 0.5 μl of each primer (diluted to a final concentration of 600 nM) and 5 μl of SYBR Green. The Real-time PCR was run with the Step One Plus system (Applied Biosystems, Foster City, CA). The hot start enzyme was activated for 20 sec at 95 °C, then the amplification was carried out using the following cycle: 95 °C for 3 sec; 60 °C for 30 sec; 40 times. After amplification, a melting curve was obtained according to the following protocol: 10 sec holding followed by a 0.05 °C increase, repeated 80 times, and starting at 55 °C. Eukaryotic translation elongation factor 1 alpha 1 (*eef1a1*), which is a suitable reference gene for quantitative gene expression studies in fish^45^ was used as an internal standard to normalize the signal (primers’ sequence for reference genes are reported in Table 2). This expression of *eef1a1* was found to be more stable than the expression of *β-actin* (CV *eef1a1* = 6% and CV *β-actin* = 8%) and was therefore preferred for normalization (as previously reported^25^).

Experiment 2: Classifying juvenile eels according to their gene expression levels.

### Animals

To test the hypothesis that a molecular sexing approach could be effective in a conservation context, we sampled eels smaller than 25 cm in two Mediterranean Lagoons, Mauguio (43°58′N, 4°03′E) and Salses-Leucate (42°87′N, 3°00′E), known to have highly skewed sex ratios. Data collected from 2011 to 2013 reported 95%, 80% and 60% of migrating silver female eels in Mauguio lagoon per year. Conversely, sampling performed during the same period in Salses-Leucate lagoon reported 12%, 5% and 5% of migrating silver female eels^46^. In June 2015, 80 eels measuring between 20 and 25 cm in total length and assumed to be still histologically undifferentiated were collected both in Mauguio and Salses-Leucate lagoons (Table 1).

Similarly to Experiment 1, eels were first anaesthetized with benzocaine (0.3 ml.l^−1^ of water) and the dose was increased up to 1.5 ml.l^−1^ of water, until the animal was killed. They were then measured to the nearest millimeter (GTCO CalComp) and weighed (Sartorius CP 153 balance, ±1 mg), before removing the gonads. The left gonad was immediately frozen in liquid nitrogen and stored at −80 °C for later RNA extraction, while the right gonad was put into Bouin’s fluid for histological analysis. Unfortunately, some right gonads were too thin and were either not collected or lost in the Bouin’s fluid (in the end, only 43 right gonads from the Mauguio group and 56 from the Salses-Leucate group were available).

### RNA extraction and reverse transcription

RNA extraction was processed as in Experiment 1. However, we performed the reverse transcription with 5 different RNA quantities, from 0.1 μg to 0.5 μg (final volume of 25 μl), because of the high heterogeneity in gonadal size (high variance in the quantity of RNA extracted). RNA was reverse transcribed using 200 U Moloney murine Leukemia virus (MMLV) reverse transcriptase (Promega). The volume of random hexamers (Promega) was adjusted with the quantity of RNA in a master mix buffer supplemented with 25 U of RNase inhibitor (RNasin; Promega). The relative numbers of samples extracted with 0.1, 0.2, 0.3, 0.4 and 0.5 μg were similar between conditions (*i.e*. in Maugio and in Salses-Leucate lagoons). Reverse transcription products were then diluted according to the volume of RNA used for the RT, to perform the quantitative real-time PCR (qPCR) as in Experiment 1. *Eef1a1* was also used as an internal standard for these samples. Comparisons of gene expression between the two populations (Mauguio and Salses-Leucate lagoons) were carried out using a linear model with mixed effects for each gene (Two plates were used to perform reverse transcription, each with 40 samples of each population, a random effect accounting for this possible difference was thus added).

### Data Analysis

Eels from Experiment 1 were first classified in groups according to their gonad histology, as previously described^25^. The Kruskall-Wallis test, followed by Wilcoxon pairwise comparison tests, were used to assess differences in gene expression between these groups. P-values were adjusted using the Holm method^47^. Large differences in the levels expressed by the genes (normalized with *eef1a1*) were observed. In order to classify individuals according to selected gonadal gene expression, we performed a discriminant analysis on standardized data [0–1] associated with a cross-validation procedure. While seven genes were considered for “adults”, only 6 were considered for “juveniles”. *Foxn5* was not considered in juveniles due to non-detection in 39 individuals. Only individuals with a level of expression detected for all of the above-mentioned genes were taken into account in the discriminant analysis, and at the end, 67 individuals were considered in the statistics of the “adult” data set and 148 individuals in the “juvenile” data sets (among which 78 were from Mauguio and 70 from Leucate). To explore differences in gene expression, nonmetric multidimensional scaling (NMDS) was used. The Bray–Curtis dissimilarity was chosen to measure differences among individuals based on standardized [0–1] expression level. A final stable solution was found after 12 random starts (Final stress = 0.12 and 0.10), respectively for juveniles and adults. Significance of the discriminant analyses was tested using the Monte-Carlo test (i.e. non parametric version of the Pillai’s test, using the *randtest* function from the *ade4* R package) using 1000 replicates.

Statistical analyses were performed using the package “lmerTest” for mixed models and the metaMDS function from the “vegan” package within the R software (R Development Core and Team, 2009).

## Results

Experiment 1: Assessing genes differentially expressed in “adults”.

### Gene expression analysis in testis, ovaries and intersex gonads

**Figure 2:**
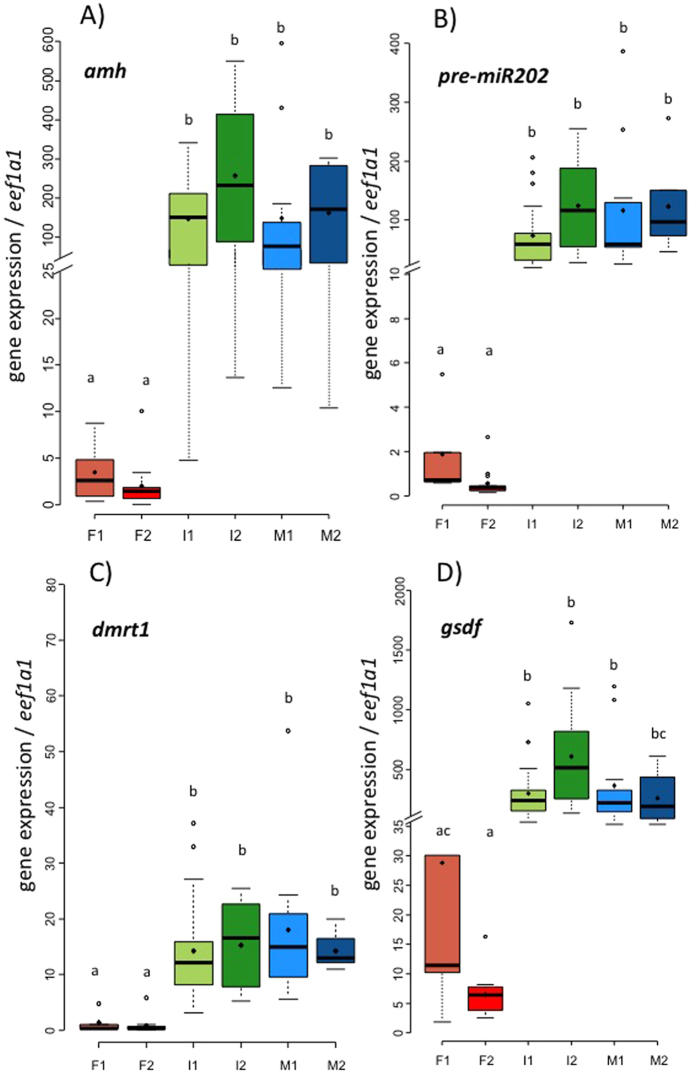
Boxplots showing the expression patterns of (**A**) amh, (**B**) pre-miR202, (**C**) dmrt1 and (**D**) gsdf relative to the expression of eef1a1 in gonads of males, females and intersexual eels at different gonadal stages. Different lowercase letters indicate significant difference (threshold p-value = 0.05) between groups. Number of samples per group: F1 = 5, F2 = 14, I1 = 19, I2 = 12, M1 = 10, M2 = 7.

The expressions of *amh* (Fig. 2A), *pre-miR202* (Fig. 2B) and *dmrt1* (Fig. 2C) were significantly higher in gonads of both intersex groups (I1 and I2) and male groups (M1 and M2) than in the gonads of both female stages (F1 and F2). The gonadal expression of *gsdf* was significantly higher in I1, I2 and M1 groups than in both female groups (F1 and F2). However, the *gsdf* expression was not significantly different between M2 and F1 (p-value = 0.07), but was significantly higher in M2 than in F2 (Fig. 2D).

**Figure 3:**
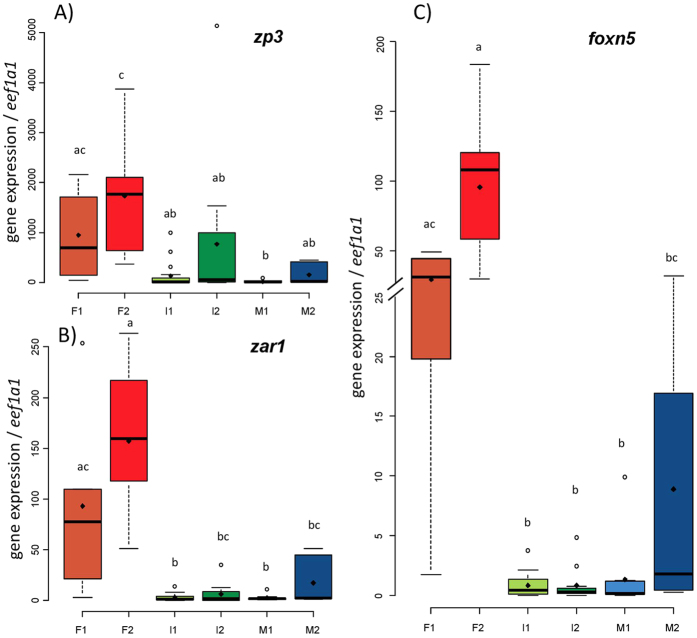
Boxplots showing the expression patterns of (**A**) *zp3*, (**B**) *zar1* and (**C**) *foxn5* relative to *eef1a1* in gonads of males, females and intersexual eels at different gonadal stages. Different lowercase letters indicate significant difference (threshold p-value = 0.05) between groups. Number of samples per group: F1 = 5, F2 = 14, I1 = 19, I2 = 12, M1 = 10, M2 = 7.

Concerning genes showing a preferential expression in females, no significant difference in their gonadal expression could be found between the two female stages, F1 and F2 (Fig. 3). mRNA levels of *zp3* were significantly higher in gonads of the F1 group when compared to the M1 group (Fig. 3A), but not when compared to I1, I2 and M2 group (Fig. 3A). The expression of *zp3* in the F2 group was significantly higher than in I1, I2, M1, and M2 groups. The expression of *zar1* and *foxn5* was significantly higher in gonads of both female group (F1 and F2) when compared to I1 and M1 group (Fig. 3B,C). For both genes, differences were not significant between F1 and M2 groups.

**Figure 4:**
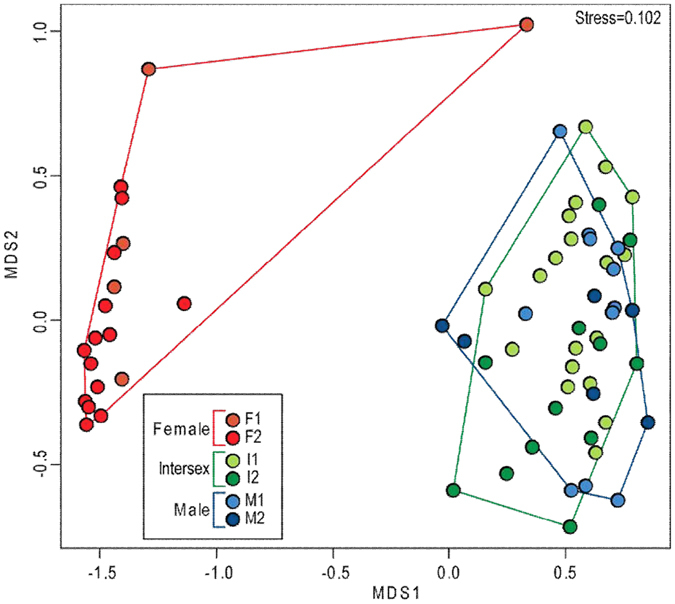
Ordination (NMDS; distance = Bray–Curtis; Stress = 0.10) of “adult” eels (N = 67) based on standardized gene expression levels of *dmrt1*, *amh*, *gsdf*, *pre*-*miR202*, *zar1*, *zp3* and *foxn5*.

The NMDS analysis of gene expression allowed good separation of females from both male and intersexual eels (Fig. 4), although these two last categories (male and intersexual) were intermingled.

The discriminant analysis was significant (3 groups: Simulated p-value < 0.001), even if the misclassification (i.e. error rate) was very high for males (Table 3A). However, when only two groups were considered (Female and Male + Intersex), misclassification was sharply reduced with error rates of only 15% and 0%, respectively for F and M + I (Table 3B).

Experiment 2: Classifying “juveniles” according to their gene expression levels.

The expressions of *zar1* and *zp3* were significantly higher in the Mauguio group (n = 80 and n = 79, respectively) when compared to Salses-Leucate group (n = 80 and n = 74, respectively). Statistics for *zar1* were: T value = 5.467, p value < 0.001 and for *zp3*: T value = 10.308, p value < 0.001. The expressions of *gsdf*, *amh* and *pre-miR202* were significantly lower in the Mauguio group (n = 80 for all 3 genes) compared to the Salses-Leucate group (*gsdf*: T value = −3.665, p value < 0.001, *amh*: T value = −3.438, p value < 0.001 and *pre-miR202*: T value = −3.300, p value = 0.0012), with n = 80 in all cases. The expression of *dmrt1* did not differ between individuals from the Mauguio lagoon (n = 79) and Salses-Leucate lagoon (n = 76), *dmrt1*: T value = 0.76, p value = 0.45.

The gonadal histology revealed that individuals from the two different locations can be classified as either undifferentiated (spermatogonia or oogonia not distinguishable at this stage), or as potentially developing females due to the presence of early oocytes. Oocytes were observable in 28% (12/43) of the Mauguio individuals and 5% (3/56) of the gonads from the Salses-Leucate lagoon.

**Figure 5:**
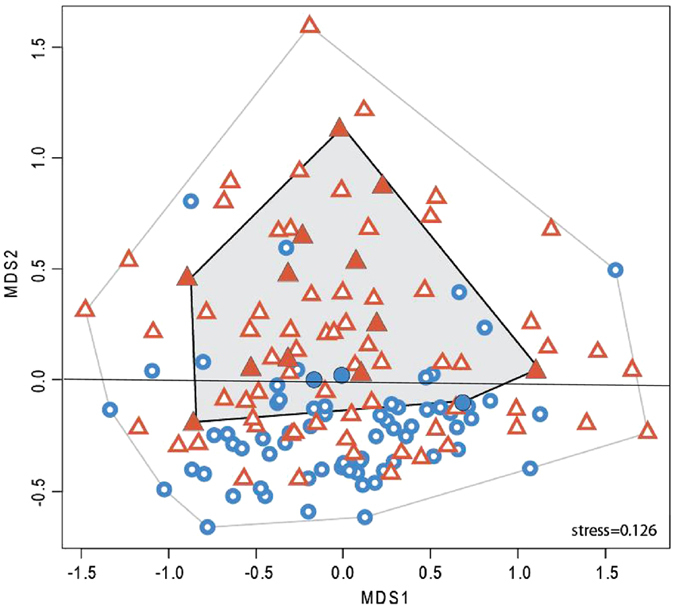
Ordination (NMDS; distance = Bray–Curtis; Stress = 0.12) of juveniles from Mauguio (triangles, N = 78) and Salses-Leucate (circles, N = 70) based on standardized gene expression levels of *dmrt1*, *amh*, *gsdf*, *pre*-*miR202*, *zar1* and *zp3*. The internal convex polygon depicts individuals histologically identified as female (full triangles or circles).

While the MDS, which was conducted on the 148 individuals for which a gene expression was observed for the all 6 genes, did not fully separate eels from the Mauguio and Salses-Leucate lagoons, individuals from the two localities tended to appear in distinct parts of the ordination plot, with those from Mauguio being mostly located in the upper part of MDS2. The related discriminant analysis was significant (Simulated p value < 0.001), and the overall correct classification rate was 68% (Table 3C). When building an area bordered by individuals with oocytes in their gonads (Fig. 5, full symbols), all eels presenting with oocytes (from Mauguio and Salses-Leucate) were inside that area. When considering the whole group, 52% of the Mauguio individuals were inside this area, whereas it concerned only a quarter of the Salses-Leucate individuals (25%).

## Discussion

To the best of our knowledge, this is the first time that the combined use of gonadal expression profiles of different genes (*dmrt1*, *amh*, *gsdf*, *pre-miR202*, *zar1*, *zp3* and *foxn5*) has been used in eels, allowing a clear and accurate discrimination of female eels from both males and intersexuals. Expression patterns support previous results^25^ which suggested that intersexual eels would not become females and that this intersexual stage should be better read as a transitional stage towards testicular differentiation. In agreement with this hypothesis, individuals from I1, I2, M1 and M2 groups were always intermingled in our analysis based on gene expression profiles, strongly suggesting that intersexual stages share a lot more similarities with males than with females.

Z*ar1* and *zp3* genes were highly expressed in ovaries when compared to both intersex and males, although this appeared to be stage-dependant with F1 females presenting similar expression levels of *zp3* and *zar1* compared to I2 and M2. Interestingly, *zp3* was also over-expressed in female Nile tilapia (*Oreochromis niloticus*) compared to males at 2 days post fecundation, 19 days before first signs of histological differentiation^48^.

In agreement with its known role on testicular differentiation (see Table 2), *dmrt1* expression in eel gonads was found to be higher in males than in females, particularly in late intersexual stage (I2) and early male sex differentiation (M1). We found that *amh* expression was 100 fold higher in intersexual and male gonads than in females, in accordance with its well described dimorphic expression in other species^36^,^49^,^50^. Like *amh* and *dmrt1*, *gsdf* was also found to act as a master sex determining gene in different species^51^,^52^. In the present study, the expression of *gsdf* was also much higher in developing male and intersexual gonads compared to females in eels. *Foxn5* was an interesting female marker based on the high level and specific expression of *foxn5* in developing ovaries. However, *foxn5* expression was frequently undetected in the gonads of juvenile eels from lagoons (not detected in 39 gonads). It may be linked both to a *foxn5* latish expression and to very low levels of expression in male gonads since 72% of the gonads without *foxn5* expression came from the male-biased population of Salses-Leucate. Our results also showed that *pre-miR202* was expressed in a strongly sexually dimorphic way, with a higher expression in testis than in ovaries. The exact function of *mir-202* is still elusive, although it has recently been shown to be regulated by *sox9* in the mouse testis^53^ and capable of influencing the expressions of *dmrt1*, *cyp19a1a* and *foxl2* in chicken gonads^41^. The only other study reporting a sexually dimorphic expression of *mir-202* in a fish species was conducted in the Atlantic Halibut (*Hippoglossus hippoglossus*)^42^, but differences in expression levels between males and females were of smaller range when compared to eels. Overall this suggests a crucial role of *mir-202* in sex differentiation^54^.

Using the combined expression of these genes, we sought to be able to efficiently estimate sex ratios. The mean expression levels for *zar1*, *zp3*, *amh*, *gsdf*, and *pre-miR202*, differed significantly between the lagoons, in accordance with our expectations. Hence, *zp3* and *zar1* were more expressed in Mauguio individuals where a higher percentage of females was expected, whereas the opposite was observed for *amh*, *gsdf* and *pre-miR202*. On average, individuals from Mauguio lagoon (sex-ratio skewed toward females) displayed lower levels for *amh*, *gsdf*, *pre-miR202* and/or higher levels for *zar1* and *zp3*, than individuals from Salses-Leucate, and the discriminant analysis was highly significant. However, it is difficult to decide where to set the limit to separate future males from future females. Depending on the method: area limited by individuals with oocytes, or positive values on MDS2 axis, molecular sex ratio assessments led to 52–63% of females in Mauguio and 25–17%, in Salses-Leucate, respectively. Even if the results from each method are very similar, a problem occurs with the border setting based on histology. In this environment where growth is very rapid, the histology might not be used because gonads with oocytes may in fact correspond to intersex gonads.

Sex-ratios reported on silver eels in the two lagoons^46^ varied between 60–95% of females in Mauguio, 5–12% in Salses-Leucate (2011–2013). However, these estimates are based on the sampling of silver eels, whereas our results are based on yellow eels. Mauguio lagoon is fed by tributaries where the proportion of females is probably quite high, in relation with the upstream location of females. Silver eels in Mauguio came from the lagoon itself and also from those tributaries. In 2009, pit-tagging of eels caught in the Mauguio lagoon and then recaptured led to an estimate of 33% of females^55^. Salses-Leucate Lagoon does not have such tributaries, and so the estimates based on silver eel catches could be similar to estimates based on juvenile catches. However, this remains true as long as mortality does not vary between sexes. Our estimate (20–25% of females in Salses-Leucate), which is double the highest percentage of female silver eels reported in that lagoon (12%)^46^ could result, at least partly, from a higher mortality rate suffered by females in relation to their longer freshwater residency before silvering (double on average). This lower mortality at silvering in males when compared to females is an expected outcome in Mediterranean lagoons where the yellow eel fishery is very active^56^. Because the temporal sex ratios may also change from year to year, further studies are needed to compare our predictions with reality. This could be achieved by sampling within the same cohort over three consecutive years and by comparing molecular sex-ratio estimates of juveniles (20–25 cm) with sex-ratio estimates based on histology of yellow eels gonads (28–45 cm) caught in the two following years.

Thanks to the combined use of several sex markers, we have showed that it is possible to differentiate male and intersex from female eels. In addition, we revealed that this method is likely to be effective at the juvenile stage when gonads are not yet histologically differentiated. Although invasive, this method would have the advantage of sampling only small individuals that are assumed to be subjected to high cumulative mortality^57^. One limitation of the proposed method relies on the fact that tiny gonads (<0.5 mm diameter) are difficult to sample. Regarding the restricted expression of the selected genes to the gonads (RNA-seq), the eels’ trunk might also be directly used for RNA extraction, although this requires further validations before being used.

ESD represents a tremendous opportunity for managers with the aim of manipulating sex-ratio to sustain critically endangered species, as has already been carried out in turtles^3^. In zebrafish, it has recently been shown that external factors triggering unbalanced sex ratio toward males, negatively affected the whole population dynamics^58^. Although such predictions still have to be demonstrated in eels, according to a previous study^14^, producing a majority of female could be an effective measure to favor population recovery. Restocking eels for conservation purpose could be a promising option for sustaining eel populations. However, this action warrants more investigation regarding the carrying capacity of the selected receiving area, since it could influence both yellow eel survival^21^,^59^ and future sex ratio^21^,^23^. The combined use of the sex markers that we propose would be useful to readily assess the population sex-ratio in a specific river/lagoon, where transplantations take place. Its broad use will enlighten the efficiency of long-term management processes and enhance the accuracy of stock modeling.

## Additional Information

**How to cite this article**: Geffroy, B. *et al*. Sexually dimorphic gene expressions in eels: useful markers for early sex assessment in a conservation context. *Sci. Rep.* **6**, 34041; doi: 10.1038/srep34041 (2016).

## Figures and Tables

**Table 1:** Numbers, mean weight and total length + (range) of eels sampled at each stage of gonadal development after rearing in the INRA facilities or collected in Mauguio and Salses-Leucate lagoons.

	Groups symbols		Undifferentiated		Females		Intersex		Males	
			U		F1	F2	I1	I2	M1	M2
Sampling period and location	February 2011	INRA Facilities	/		1	3	5	11	2	/
	July 2011	INRA Facilities	/		4	11	14	1	8	7
	June 2015	Mauguio		80	/	/	/	/	/	/
	June 2015	Salses-Leucates	80		/	/	/	/	/	/
Mean Weight (g) and range			20 (12–29)	17 (9–28)	83 (68–98)	143 (52–348)	103 (68–155)	95 (65–116)	81 (59–114)	103 (93–109)
Mean Size (cm) and range			244 (206–282)	226 (195–264)	368 (347–386)	419 (321–535)	377 (336–440)	364 (335–398)	346 (330–388)	375 (352–394)

Gonadal stages are defined in the reference^25^. Eels sampled in 2011 were also used for the experiment conducted in the reference^25^. The same individuals were used for qPCR. Among the 160 individuals from lagoons, not all were considered in the statistics due to NA value for some genes (see text for exact numbers).

**Table 2:** Name and known function of genes for which specific primer pairs have been designed for quantitative real-time PCR.

Gene name	Gene symbol	Primers (5′–3′)	Sex det.	Sex diff.	Gam.	Male/Female
Eukaryotic translation elongation factor 1 alpha 1	*eef1a1*	ATTGTGGGAGTCAACAAGATGGAa GCTGACTTCCTTGGTGATTTCCTb	/	/	/	both
β-Actin	*βact*	AATCCACGAGACCACCTTCAACTa TGATCTCTTTCTGCATTCTGTCGb	/	/	/	both
forkhead box N5	*foxn 5*	CCTCGTCCAGCGAATATCTTCTTa TGTTTTGAGCGAGATTCAGCTTCb		?^39^,^40^,^61^		both*
Zygotic arrest 1	*zar1*	TGAGGTTTCAGTTCTTGGAGCAGa TAAACCTTGTTGGTTCCCTGGACb			✓^30^	Female
Zona pellucida 3	*zp3*	GCAATGACCGAAGACTCCCTAGTa CGTTGTGTAGCCTCAGGTAATGGb			✓^31^,^60^	Female
doublesex and mab-3 related transcription factor 1	*dmrt1*	ATCTGCAGTCCAGTCACCTTGTCa GTTTTGGAGAGGGGGAGACTTTTb	✓^32^	✓^63^,^33^	✓^34^	Male
gonadal somatic cell derived factor	*gsdf*	AAAGCGGTCTTAGGAGACACTGCa CTGCAAGCCTGTACTGTTGGTCTb	✓^51^	✓^38^	✓^38^	Male
Pre-microRNA 202	*pre-miR202*	TACAGCCCCATTTTCCCATAa CAGGACTCGCTCTTCCTTTTb		?^42^,^53^	?	Male
anti-Mullerian hormone	*amh*	GATCTGACCCGTTTCGGACTTT_a_ TTAGTGGGGTGTAAAACGTGCAGb	✓^62^	✓^49^,^50^,^63^	✓^50^	Male

Sex det.: Sex determination, Sex diff.: Sex differentiation, Gam.: Gametogenesis (refers to the development of the ovary or the testis). Male/Female: indicate whether the gene has been found predominantly expressed in ovary or in testis by multi-tissue RNA-seq analysis. Numbers^30^,^31^,^32^,^33^,^34^,^38^,^39^,^40^,^42^,^50^,^53^,^61^,^62^,^63^ correspond to Article numbers in the bibliography. “?” Indicates that the role is still unknown. Both *eef1a1* and *β-actin* were initially used as reference genes, but since *eef1a1* was more stable it was used subsequently. *Foxn5 is rather reported to be involved in embryogenesis. See discussion for a more complete description of the role of each gene.

**Table 3:** Confusion matrix depicting performance of discriminant analysis for A/adults (Female, Male, and Intersex) B/adults (Female, Male + Intersex) C/juveniles (Mauguio, Salses-Leucate).

**A)**	Determined as/predicted as	Female	Male	Intersex	Error (%)
	Female	17	0	2	10.5
	Male	0	2	15	88.2
	Intersex	0	6	25	19.3
**B)**	Determined as/predicted as	Female	Male + Intersex	Error (%)	
	Female	16	3	15.7	
	Male + Intersex	0	48	0	
**C)**	Originating from/predicted from	Mauguio	Salses-Leucate	Error (%)	
	Mauguio	45	33	42.3	
	Salses -Leucate	14	56	20	

Each column represents predicted class while each row represents true class. Misclassification (error rate) is provided for each row.
